# Mannose Receptor-Mediated Carbon Nanotubes as an Antigen Delivery System to Enhance Immune Response Both In Vitro and In Vivo

**DOI:** 10.3390/ijms23084239

**Published:** 2022-04-11

**Authors:** Haibo Feng, Yangyang Feng, Lang Lin, Daiyan Wu, Qianqian Liu, Hangyu Li, Xinnan Zhang, Sheng Li, Feng Tang, Ziwei Liu, Linzi Zhang

**Affiliations:** 1 College of Animal Husbandry and Veterinary Medicine, Southwest Minzu University, Chengdu 610041, China; f1733678933@126.com (Y.F.); wdx1063196822@126.com (D.W.); 15892603728@163.com (Q.L.); li1998hangyu@163.com (H.L.); n1547187363@163.com (X.Z.); listen699@163.com (S.L.); tangfeng1719556170@163.com (F.T.); z17393117167@163.com (Z.L.); zlz754130837@163.com (L.Z.); 2 Key Laboratory of Ministry of Education and Sichuan Province for Qinghai-Tibetan Plateau Animal Genetic Resource Reservation and Utilization, Chengdu 610041, China; 3 College of Veterinary Medicine, Southwest University, Rongchang, Chongqing 402460, China; umbrella_fj@163.com

**Keywords:** mannose receptors, mannose-modified carbon nanotubes, antigen delivery system, immune response

## Abstract

Carbon nanotubes (CNTs) are carbon allotropes consisting of one, two, or more concentric rolled graphene layers. These can intrinsically regulate immunity by activating the innate immune system. Mannose receptors (MR), a subgroup of the C-type lectin superfamily, are abundantly expressed on macrophages and dendritic cells. These play a crucial role in identifying pathogens, presenting antigens, and maintaining internal environmental stability. Utilizing the specific recognition between mannose and antigen-presenting cells (APC) surface mannose receptors, the antigen-carrying capacity of mannose-modified CNTs can be improved. Accordingly, here, we synthesized the mannose-modified carbon nanotubes (M-MWCNT) and evaluated them as an antigen delivery system through a series of in vitro and in vivo experiments. In vitro, M-MWCNT carrying large amounts of OVA were rapidly phagocytized by macrophages and promoted macrophage proliferation to facilitate cytokines (IL-1β, IL-6) secretion. In vivo, in mice, M-MWCNT induced the maturation of dendritic cells and increased the levels of antigen-specific antibodies (IgG, IgG1, IgG2a, IgG2b), and cytokines (IFN-γ, IL-6). Taken together, M-MWCNT could induce both humoral and cellular immune responses and thereby can be utilized as an efficient antigen-targeted delivery system.

## 1. Introduction

Carbon nanotubes (CNTs), a synthetic allotrope of carbon, are composed of one (single wall), two (double wall), or more (multi-wall) concentric rolled graphene layers [1,2]. With the cylindrical shape and chemical structure (honeycomb carbon lattice), CNTs offering a delocalized pi-electron cloud and high surface area to volume ratio have great potential in biomedicine, such as a drug carrier [3,4]. CNTs are a novel carrier system that can specifically and effectively deliver therapeutic drugs to the required target site [5,6,7]. Moreover, owing to their unique properties, CNTs have emerged as an exciting tool for treatment and diagnosis in the medical field. Notably, CNTs can also activate the innate immune system [8]. Salvador-Morales et al. were the first to report complement activation by nonfunctionalized CNTs [7]. Both primitive and functionalized CNTs can be effectively phagocytosed by macrophages in vitro, therefore, CNTs having inherent adjuvant effects can be promising antigen carriers [9,10]. Moreover, these can induce various genes and nuclear factors, complement proteins, and cytokines production [11,12]. 

Antigen-presenting cells (APCs) play a vital role in the cellular immune response by absorbing, processing, and then transporting antigens to the cell surface via major histocompatibility complexes (MHC-I or II) [13,14]. The physical properties of CNTs allow them to readily enter the APCs and stimulate the expression of MHC-II and MHC-I complexes to induce immune responses [15,16]. Besides, CNTs have good adsorption properties, especially for protein molecules. Moreover, connecting protein molecules to CNTs reduces their possible toxicity which can be advantageous as vaccine carriers [16,17]. Pantarotto D et al. showed that the CNT-attached viral envelope peptide epitope structure remained immunogenic, suggesting that the method can enhance virus-specific neutralizing antibody responses [18]. Silvestre et al. investigated the humoral and immune cellular responses in calves immunized with carbon nanotubes and rMSP1a (N-terminal repeat of the protein of *A. marginale* strain) (MWNT+rMSP1a) and found the total number of NK cells, leukocytes, and the levels of IgG, IgG1, and IgG2 antibodies were significantly increased [19]. This showed that MWCNTs can promote antigenic efficiency of the carrier to strongly induce cellular and humoral immune responses.

The mannose receptor (MR), belonging to the C-type lectin superfamily and mainly expressed by macrophages and dendritic cells (DCs), is involved in the homeostasis process and pathogen recognition [20,21]. It has three different extracellular binding sites that can recognize both endogenous and exogenous ligands to induce antigen uptake by APCs [20,21]. In addition, the MR can recognize mannose or mannose conjugated drug delivery materials or adjuvant [22,23,24]. Mannose, a widely distributed monosaccharide in tissues and body fluids, is an important tool in pharmacotherapy. It can be utilized for immune regulation and glycoprotein synthesis [25]. Moreover, as a vaccine adjuvant, mannose can improve the internalization and expression of the related co-stimulatory molecules. For these reasons, mannose-modified drugs or delivery materials have become a popular method for targeted drug delivery via mannose receptors [22,23,24]. Goswami et al. used mannose-cholesterol amine-conjugated mannosylated lipid nanoparticles (MLNP) to deliver influenza (hemagglutinin) encoded SAM vaccine in mice and found that the carrier enhanced the in vitro antigen uptake by bone marrow-derived dendritic cells and in vivo antigen-specific CD4+ and CD8+ T cell responses, indicating excellent antigen delivery efficiency with augmented immune response [26]. Likewise, Min et al. showed that mannose-modified nanoparticles (NPs) can better capture the antigens to present them to dendritic cells [27]. Yang et al. constructed a cancer vaccine using mannose-modified cancer cell membranes by encapsulation in immune-adjuvant nanoparticles. They found that the efficacy of the NPs significantly improved and mannose receptor target modification increased antigen uptake in dendritic cells [28]. 

In this study, we used mannose-modified MWCNT to design a mannose receptor-targeted delivery system for effective antigen delivery to macrophages or DCs. M-MWCNT was synthesized and its physicochemical properties and targeting were investigated both in vitro and in vivo. We found that M-MWCNT significantly improved antigen delivery to macrophages and promoted immune activity in vitro. Moreover, it effectively decreased the release rate of antigen, and dramatically enhanced the in vivo humoral and cellular immune responses against OVA. Taken together, we suggest that M-MWCNT can be an efficient targeted antigen delivery system.

## 2. Materials and Methods

### 2.1. Reagent and Cell Line

Carboxylic carbon nanotubes (C-MWCNT), mannose, complete Freund’s adjuvant, ovalbumin, and concanavalin A were purchased from Sigma-Aldrich (Nanocyl Inc., Sambreville, Belgium). Cytokine ELISA kits and fluorescently labeled monoclonal antibodies for FCM (flow cytometry) were purchased from Seymour Fisher Technology Co., Ltd., IgG and IgG subtype antibodies were purchased from SANTA CRUZ Co., Ltd. (Shanghai, China). 4-dimethylaminopyridine (DMAP) and 1-Ethyl-3-(3-dimethy laminopropyl) carbodiimide hydrochloride (EDC) were purchased from Yuanye Bio-Technology Co., Ltd. (Shanghai, China). ACK Lysis Buffer was purchased from Becton Dickinson Co., Ltd. (Shanghai, China). Cell Counting Kit-8 (CCK-8) was obtained from the Beyotime Institute of Biotechnology (Jiangsu, China). Penicillin-Streptomycin Liquid and Trypsin-EDTA solution were purchased from Beijing Solarbio Science & Technology Co., Ltd. (Beijing, China). Mouse Macrophage RAW264.7 Cells lines, RPMI-1640, and DMEM were obtained from Shanghai Zhongqiao Xinzhou Biological Technology Co., Ltd. (Shanghai, China). CH_2_CL_2_ was purchased from Chongqing Chuandong Chemical (Group) Co., Ltd. (Chongqing, China). Fetal Bovine Serum was obtained from Zhejiang Tianhang Biological Technology Co., Ltd. (Hangzhou, Zhejiang, China). The Bicinchoninic acid (BCA) Protein Assay Kit was obtained from Nanjing Jiancheng Biological Technology Co., Ltd. (Jiangsu, China).

### 2.2. Preparation of M-MWCNT

**Figure 1:**
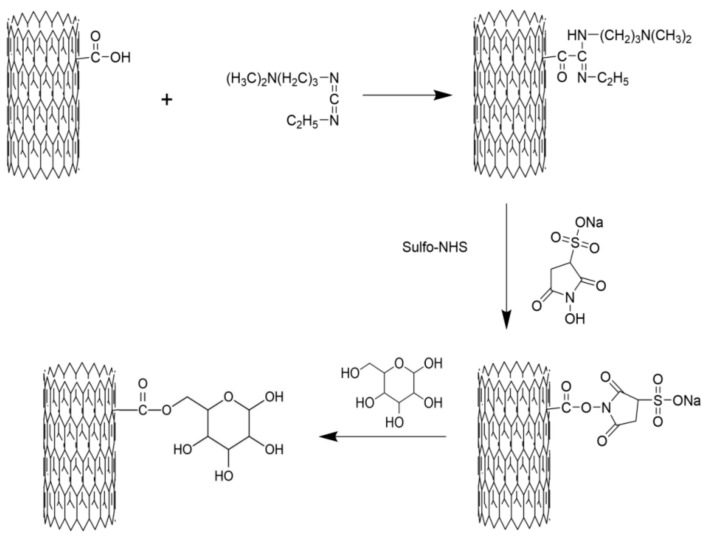
Schematic diagram of preparing the mannose-modified carbon nanotubes antigen delivery system.

One gram of C-MWCNT and 30 mL of CH_2_CL_2_ added in a tube were mixed by crushing using 200 W ultrasonication for 30 min. Then, 150 mg of EDC and 30 mg of DMAP were added and the mixture was incubated for 20 min to activate the C-MWCNT. Subsequently, 130 mg of mannose was added to the mixture and the reaction was performed at 40 °C for 12 h. Next, the reaction mixture was centrifuged at 8000 rpm for 10 min, the supernatant was discarded, and the precipitate was washed 5 times with deionized water. The formed CNTs were re-suspended to dispersed in 30 mL of deionized water and dialyzed against distilled water in a 20,000 Da cut-off dialysis sack for 7 days. Lastly, the dialyzed product was lyophilized to obtain M-MWCNT using a vacuum lyophilizer (Ningboxinzhi, SJIA-18N, Ningbo, China). To obtain M-MWCNT+OVA, ovalbumin (OVA) protein was loaded onto the surface of M-MWCNT via physical adsorption. Briefly, 1 mg of M-MWCNT was uniformly dispersed in 10 mL of normal saline by ultrasonication for 20 min. Then, 1 mg of OVA was added and mixed by stirring at 40 °C (Shanghai Lichen, Shanghai, China). 1 mL of OVA treated mixture was taken out at 30, 60, 90, 120, 150, and 180 min, and centrifuged at 10,000 rpm for 10 min. The OVA concentration in the supernatant was detected using the BCA kit. The schematic diagram of preparing the mannose modified carbon nanotubes is shown in Figure 1.

### 2.3. Characterization of M-MWCNT Composites

**Transmission electron microscopy (TEM):** The morphological examination of the M-MWCNT was conducted using TEM (TEM, JEM-1200, Tokyo, Japan).

**Fourier transform infrared (FTIR) spectroscopy analysis:** The CNTs, mannose, and mannose–CNTs were put into a mortar, respectively, and evenly mixed with ~15 mg of spectral pure KBr powder by grinding. At 15 T pressure compression, the characteristic infrared spectra were recorded at 4000–400 cm^−1^ by an FT-IR spectrometer (Nicolet iS50, Thermo Fisher Scientific, Cleveland, OH, USA).

**Ultraviolet spectrum (UV) analysis:** An equal concentration of CNTs, mannose, mannose–CNTs was evenly dispersed in deionized water, respectively, and the solution was exposed to 195–250 nm ultraviolet light to record the absorbance by a UV-5100 spectrophotometer (Yuanxi Instrument Co., Ltd., Shanghai, China).

**Raman spectroscopy (RA):** The C-MWCNT, M-MWCN, and mannose powders were analyzed by Raman spectrometry (Horiba, LabRAM HR, Villeneuve d’Ascq, France) with an excitation wavelength of 532 nm. The samples were deposited on glass slides and detailed scans were recorded in the range of 1000–2000 cm^−1^.

**X-ray photoelectron spectroscopy (XPS):** The surface properties were evaluated by X-ray photoelectron spectroscopy (AXIS Supra, Kratos, UK).

**Thermogravimetric analysis (TGA):** The samples were subjected to thermogravimetric analysis (SDT650, Saugus, MA, USA). Briefly, 10 mg of C-MWCNT, 10 mg of mannose, and 10 mg of M-MWCNT were heated in flowing nitrogen at 20 °C/min (20–800 °C) to automatically record the TG curve by software. Data were processed and the lost weight of C-MWCNT, mannose, and M-MWCNT was calculated to estimate mannose grafting by using the following formula:

Grafting rate % = (M-MWCNT thermal weight loss percentage − C-MWCNTs thermal weight loss percentage)/mannose thermal weight loss percentage × 100%.

**Zeta potential analysis:** The surface charge of a CNT directly affects its stability. The potential of CNTs can be estimated by measuring the electromotive force of surface colloidal particles. Zeta potential values of C-MWCNT, M-MWCNT, and M-MWCNT+OVA in 10 mM of PBS were measured by a nanoparticle size potential analyzer (Anton Paar, Litesizer 500, Vienna, Austria) at 25 °C in pH 7.4.

**Antigen adsorption assay:** 1 mg of M-MWCNT was evenly dispersed in 10 mL of normal saline by 200 W ultrasonication for 20 min. Then, 1 mg of model antigen (OVA) was added using stirring at 40 °C. One milliliter of M-MWCNT samples was collected at 30, 60, 90, 120, 150, and 180 min, respectively. The supernatant was centrifuged at 12,000 rpm for 10 min and the OVA concentration in the supernatant was estimated by the BCA kit.

### 2.4. In Vitro Test

#### 2.4.1. Cytotoxicity on Macrophages

The M-MWCNT cytotoxicity on macrophages was studied as described previously [29]. Briefly, the macrophages were cultured for at least 3 generations to grow well. Then, 100 μL of macrophage suspension (4 × 10^5^ cells) was plated into a 96-well plate. One-hundred microliters of cell culture medium containing different concentrations of M-MWCNT was added to each well to reach a final concentration of 0 (blank control), 10, 20, 30, 40, and 50 μg/mL M-MWCNT, respectively. The mixture was incubated at 37 °C in a 5% CO_2_ incubator for 44 h. Then, 20 μL of CCK-8 solution was added to each well and further incubation was performed for 4 h. The absorbance at 450 nm was measured by a microplate reader (iMark, Bio-Rad, Hercules, CA, USA) and the most suitable drug concentration was found from the following cell activity formula as stated in the CCK-8 kit:

$$\mathrm{Cell}\mathrm{viability}\;(\%)=\frac{\left(A_{S}-A_{B}\right)}{\left(A_{Z}-A_{B}\right)}\times 100\%$$

where A_S_: with cells, CCK-8 solution absorbance of drug wells; A_B_: absorbance of wells with medium and CCK-8 solution without cells; A_Z_: absorbance of wells with cells and CCK-8 solution without drugs.

#### 2.4.2. Effects of M-MWCNT on Cytokine Secretion of Macrophages

The cell concentration was adjusted to 4 × 10^6^/mL per well. A relatively non-toxic concentration was selected based on the CCK-8 cytotoxicity test (30 μg/mL). Macrophage cells suspension was seeded into a 12-well plate (500 μL/well) and 500 μL of M-MWCNT (60 μg/mL), C-MWCNT (45 μg/mL), mannose (15 μg/mL), LPS (5 μg/mL), CFA (10 μg/mL), and DMEM was added, respectively. In each group, four wells were used as replicates. After incubation at 37 °C in 5% CO_2_ for 48 h, the supernatant was collected and centrifuged at 1500 rpm for 5 min. The supernatant concentrations of IL-6 and IL-1β in the macrophages culture were estimated by an ELISA kit (Thermo Fisher Scientific, Cleveland, OH, USA)

#### 2.4.3. TEM of Macrophages

The macrophages in the logarithmic growth phase were incubated at 37 °C in 5% CO_2_ for 12 h. After the cells were attached, the M-MWCNT solution was added and the culture was continued for another 12 h. Then, the culture medium was poured out, and 1 mL of trypsin solution was used to gently wash the culture flask to remove all the cells. To stop the elution, 4 mL of DMEM medium was added. The collected cell suspension was centrifuged at 1000 rpm for 5 min, washed twice with PBS, and the cell mass was immediately fixed with 2.5% glutaraldehyde. The fixed cells were sectioned and observed by transmission electron microscope (TEM, JEM-1200, Tokyo, Japan).

#### 2.4.4. Antigen Internalization Assessment of RAW264.7 by CLSM

Macrophages were cultured in a 6-well plate (Costar) with a round coverslip for 24 h. Then, M-MWCNT+FITC-OVA or FITC-OVA was added and the culture with RAW264.7 was continued for another 24 h. Then, the treated cells were fixed with paraformaldehyde (4%) for 15 min at 4 °C and stained with DAPI for 5 min. Lastly, the cells were mounted with glycerol (90%) and observed by a confocal laser scanning microscope (CLSM, LSM 800, ZEISS, Jena, Germany).

### 2.5. In Vivo Test

#### 2.5.1. Mice Grouping and Treatment

One-hundred and forty-four female ICR mice (weight: 18–22 g; age: 5 weeks, Grade II) were purchased from Chongqing Tengxin Bio-Technology Co., Ltd. (Chongqing, China). The lab animal procedures were performed according to international principles, mentioned in the government of China issued Guidelines for Keeping Institutional Animals Care and Use Committee (IACUC) of Southwest University (IACUC-20191216-11). The animals were randomly divided into 6 groups (each = 24) and after one-week adaptation, these were subcutaneously inoculated with 0.2 mL of OVA (15 µg/mL), M-MWCNT (30 µg/mL), C-MWCNT (22.5 µg/mL), mannose (7.5 µg/mL), physiological saline, and CFA as adjuvants. Saline alone was injected as the blank control group. Inoculation was performed three times once a week. The mice were sacrificed on days 7, 14, 21, and 28 after the first vaccination. The collected serum samples were stored at −80 °C after centrifugation at 4000 rpm for 10 min at 4 °C. The spleens were removed to prepare the single-cell suspension for further analysis.

#### 2.5.2. Analysis of Activation and Maturation of Splenic Dendritic Cells

After 48 h of the first immunization, 3 mice in each group were randomly selected to prepare a splenocyte suspension (2 × 10^6^ cells/200 μL). The cells were blocked by 5% skim milk for 30 min at 4 °C. Subsequently, the splenocytes were stained with respective fluorescent antibodies (anti-CD11c-FITC, anti-MHC-II (I-A/I-E)-PE, anti-CD86-PE, anti-CD80-PE, and isotype controls) by shaking for 30 min in an ice bath and dark environment. The reaction was terminated with 1.8 mL PBS and the supernatant was removed after centrifugation (1500 rpm, 5 min). The cells were washed twice with PBS and then suspended in 1 mL PBS. The cells were screened with a 200-mesh monolayer nylon net and the cell suspensions were collected in the flow cell tube for flow cytometry (BD FACSVerseTM, San Jose, CA, USA). The FITC and PE double-positive cells were identified as DCs that expressed surface molecules. For each splenocyte sample, the percentage of FITC and PE double-positive cells was analyzed against the total cells.

#### 2.5.3. The Effect of M-MWCNT on Proliferation of Splenocytes in Mice

After immunization, the spleens of mice were removed on days 7, 14, 21, and 28 to prepare the single-cell splenocyte suspensions (1 × 10^7^ cells/mL) as described previously [29]. The cell suspension (100 μL per well) was added to the cell culture plate (Costar) along with 100 μL of LPS solution (final concentration 10 μg/mL), or 5 μg/mL (final concentration) of Con A. The plate was cultured for 48 h at 37 °C and 5% CO_2_ while 20 μL of CCK-8 solution was added at 44 h of the culture. Lastly, the absorbance was recorded at 450 nm by a microplate reader (iMark, Bio-Rad, Hercules, CA, USA). Spleen lymphocyte proliferation ability = OD value experimental well/OD value control well).

#### 2.5.4. The Effects of M-MWCNT on the Serum Contents of IFN-γ and IL-6 in Mice

The serum concentrations of IFN-γ and IL-6 were determined by an ELISA kit (Thermo Fisher Scientific, Cleveland, OH, USA) following the manufacturer’s instructions on days 7, 14, 21, and 28 after the primary vaccination. The absorbance was detected at 450 nm and all experiments were performed in quadruplicate.

#### 2.5.5. The OVA-Specific Antibody Titer in the Mice Serum

On days 7, 14, 21, and 28 after the primary immunization, the collected mouse serum was diluted (100 times) with PBS for analysis. The final OVA concentration was adjusted to 5 μg/mL with 0.01 mol/mL of carbonate buffer. One-hundred microliters of OVA solution was added to each well in a 96-well ELISA plate and incubation was performed for 18 h at 4 °C. The plate was washed thrice. Then, 100 μL of diluted IgG antibodies was added for incubation at 25 °C for 1 h. Next, after washing thrice with scrubbing solution, 100 μL of TBM substrate chromogenic solution was added and incubation was performed in the dark for 10 min. Lastly, the OD values were determined at 450 nm. The detection methods of IgG1, IgG2a, and IgG2b were the same as above, and all experiments were performed in quadruplicate.

#### 2.5.6. In Vivo Antigen Release Assay

The release effect of model antigen (OVA) in M-MWCNT+OVA was measured in ICR mice. The mice were randomly divided into M-MWCN+OVA, C-MWCN+OVA, and OVA groups, respectively. The antigen release assays were evaluated through an in vivo optical imaging system (IVIS Lumina III^®^, Perkin Elmer™). The OVA was labeled using a Cy5.5 fluorescent dye according to the manufacturer’s protocol. A 0.2 mL aliquot of differing vaccine formulations (20 μg Cy5.5/mice) was inoculated into the lateral thigh muscle of individual mice. The fluorescence intensities in the body at 24, 48, 72, 168, and 336 h post-injection were determined, and the spleen, liver, heart, kidney, and lung of injected mice were removed at 24, 168, and 336 h post-injection, and the fluorescence intensity was detected by the fluorescence imaging system and analyzed using IVIS Lumina^®^ Software.

#### 2.5.7. Histological Toxicity Analysis of M-MWCNT

On the 28th day after immunization, tissues from major organs (heart, liver, spleen, kidney, and lung) were harvested from each group and histological analysis was performed.

#### 2.5.8. Statistical Analysis

Excel software was used for data collection and analysis. Data, expressed as the mean ± SD, were analyzed by the SPSS software (SPSS 20.0, Chicago, IL, USA). The data were analyzed by one-way ANOVA and then Duncan’s multiple-range tests. *p* < 0.05 denotes statistical significance and is indicated by different letters (a–e).

## 3. Results

### 3.1. Characterization of M-MWCNT

#### 3.1.1. TEM Morphologies of M-MWCNTs

**Figure 2:**
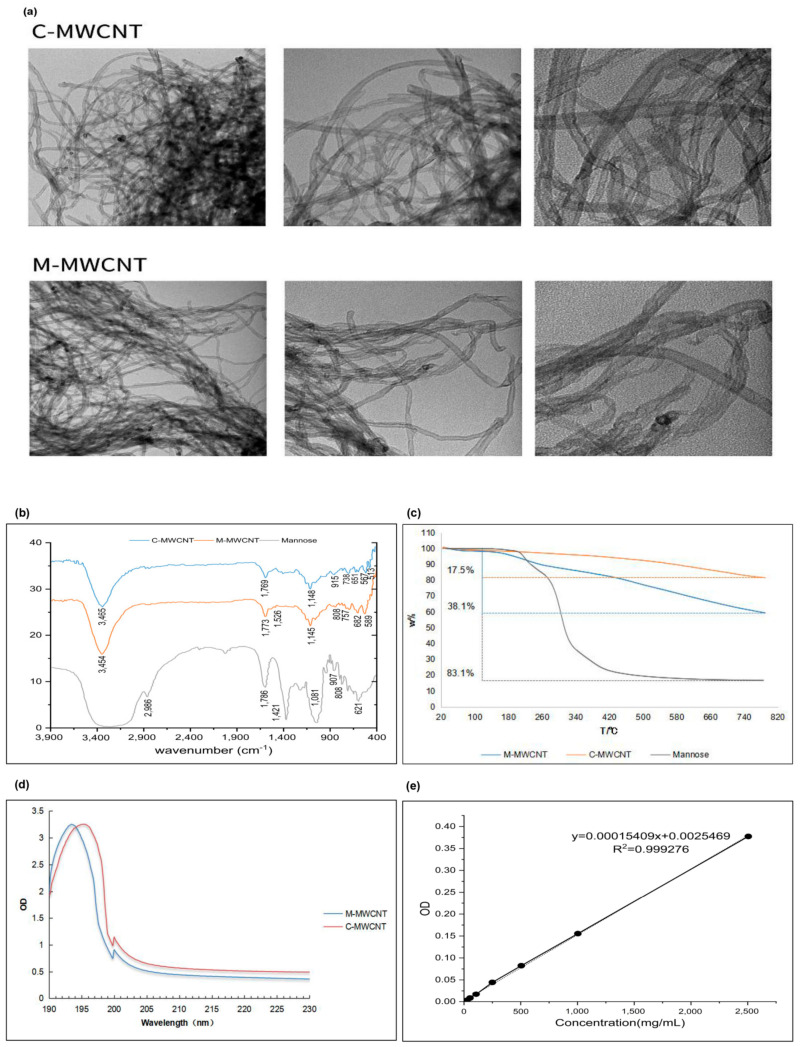

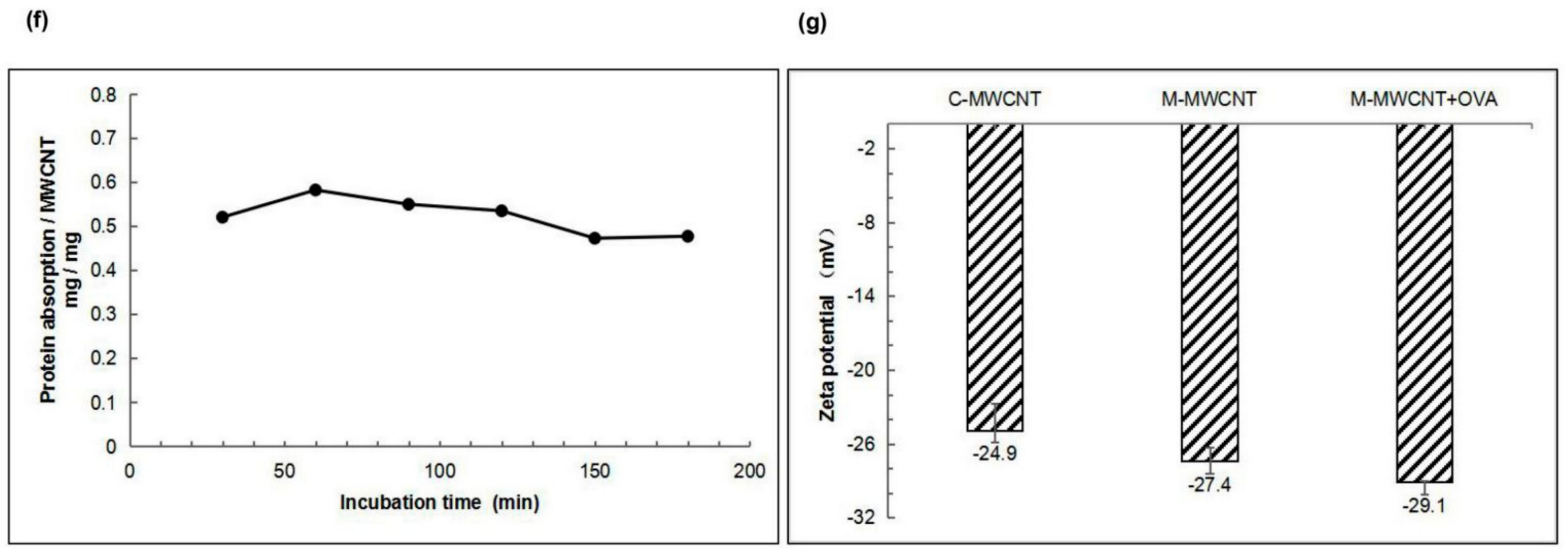
Functionalization and physicochemical characterization of M-MWCNT. (**a**) TEM images of C-MWCNT and M-MWCNT. (**b**) FTIR transmission spectra of C-MWCNT, M-MWCNT, and Mannose. (**c**) TGA curves of C-MWCNTs, M-MWCNT, and mannose. (**d**) UV absorbance spectra of C-MWCNT and M-MWCNT. (**e**) Standard curve of OVA adsorption. (**f**) Kinetics of OVA adsorption on M-MWCNT. (**g**) Zeta potential of M-MWCNT. (*p* < 0.05).

The morphologies of M-MWCNTs and C-MWCNTs were observed by TEM, and the representative images are shown in Figure 2a. We observed that the CNTs were hollow with a diameter of ~10 nm and length of ~2 μm. The unfunctionalized CNTs were distinctly aggregated and their water dispersion improved after mannose modification.

#### 3.1.2. FT-IR Spectroscopy

The FT-IR spectra of mannose, M-MWCNTs, and C-MWCNTs were analyzed within the range 4000–400 cm^−1^ (Figure 2b). The C-MWCNT exhibiting absorption peaks at 3471, 1650, and 1150 cm^−1^ corresponded to -OH, C-C, and C-O-C, respectively. The wide and strong absorption peak of mannose at 3390 cm^−1^ is for the stretching vibration of -OH. Moreover, the absorption peak at 2960 cm^−1^ represents C-H vibration, while the deformation absorption peak at 1409 cm^−1^ represents CH_2_. The bending vibration at 1650 cm^−1^ corresponds to the -OH, while the peaks at 1089, 879, and 808 cm^−1^ represent alcohol hydroxyl and other characteristic absorption peaks of mannose [30]. The infrared spectrum of M-MWCNTs revealed new peaks at 1649, 1598, 1548, and 1008 cm^−1^ which indicate the ester bond formation between the mannitol hydroxyl group and the carboxyl group of the CNTs. Moreover, the new peaks at 879 and 804 cm^−1^ signify the existence of mannose in M-MWCNTs.

#### 3.1.3. UV Spectrum Analysis

The UV spectrum analysis of M-MWCNT and C-MWCNT is shown in Figure 2c. Notably, after mannose modification, the UV absorption spectra of M-MWCNT showed a redshift from 193.7 to 195.4 nm. This could be due to the electronic transition after the formation of the carbonyl bond.

#### 3.1.4. Thermogravimetric Analysis

The thermogravimetric analysis is shown in Figure 2d. The thermal weight loss of the sample was calculated under nitrogen at 20–800 °C. In the temperature range of 130–800 °C, the total weight loss of mannose, C-MWCNT, and M-MWCNT was 83.1%, 17.5%, and 38.3%, respectively. The grafting rate of mannose was 25%.

#### 3.1.5. OVA Adsorption of Mannose Carbon Nanotubes

As shown in Figure 2e, the OVA concentration (x) and OD value (y) showed a good linear relationship, expressed as: y = 0.00015049x + 0.0025469, R2 = 0.99927. The OVA adsorption capacity of M-MWCNT was calculated using a standard curve. We found that OVA adsorption peaked (58% of the M-MWCNT mass) when it reacted with C-MWCNT for 60 min. However, with a further increase in reaction time, the adsorption capacity decreased maintaining the mass ratio of 50% after 150 min (Figure 2f).

#### 3.1.6. Zeta Potential Analysis

The zeta potentials of M-MWCNT, C-MWCNT, and M-MWCNT are shown in Figure 2g. After mannose modification, the zeta potential of M-MWCNT decreased from −24.9 ± 2.2 mV to −27.4 ± 1.1 mV and then changed to −29.1 ± 0.1 mV after OVA adsorption, indicating good stability of M-M

#### 3.1.7. XPS Analysis

**Figure 3:**
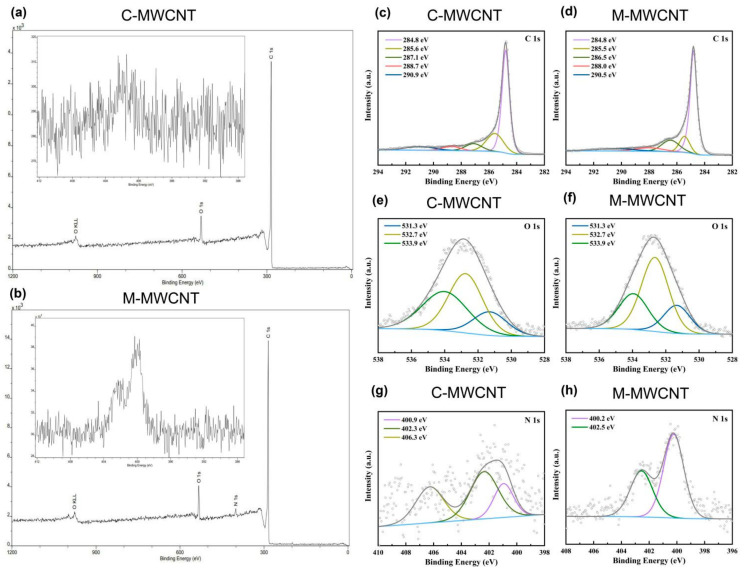
XPS analysis of the M-MWCNT and C-MWCNT: (**a**,**b**) XPS survey scan spectra of M-MWCNT and C-MWCNT. The inset is magnification of the N1s region in M-MWCNT and C-MWCNT spectrum. (**c**,**d**) Fitted XPS C1s spectra of M-MWCNT and C-MWCNT. (**e**,**f**) Fitted XPS O1s spectra of M-MWCNT and C-MWCNT. (**g**,**h**) Fitted XPS N1s spectra of M-MWCNT and C-MWCNT.

Elemental analysis results indicated that C, O, and N elements in M-MWCNT were 90.97%, 6.82%, and 2.21% by weight, respectively. The characteristic peaks of O1s and C1s photoelectrons in the scanning spectra of M-MWCNT- and C-MWCNT are illustrated in Figure 3a,b. Deconvolution of high-resolution XPS spectra over the C1s region for nanotubes is shown in Figure 3c,d. The five individual component peaks of C1s spectra represent carbon in graphite at 284.8 eV, the peaks at 285.5 eV and 286.5 eV represent carbon in aliphatic structures, and in alcohol, ether, and phenolic groups, respectively. The peak at 288.0 eV corresponds to carbon in lactone, ester, or carboxyl groups, and satellite peaks at 290.5 eV represent π–π* transitions in aromatic rings (π–π* transition). As shown in Figure 3d,e, the O1s spectrum of the nanotubes was deconvoluted into three bands—carbonyl oxygen atoms at 531.3 eV, carbonyl oxygen atoms in carboxyls and amides at 532.7 eV, and oxygen atoms in hydroxyls or ethers at 533.9 eV. In addition, a small peak of N1s photoelectrons (N elements 2.21% by weight) was also observed in the M-MWCNT spectrum, while the corresponding area of C-MWCNT showed noise signals (N elements 2.21%) (Figure 3b). Furthermore, multiple noise spots were observed in the N1s spectra of C-MWCNT and Man-MWCNTs indicting trace amounts of N, which is consistent with the result of the element analysis (Figure 3g,h).

#### 3.1.8. Raman Spectra Analysis

**Figure 4:**
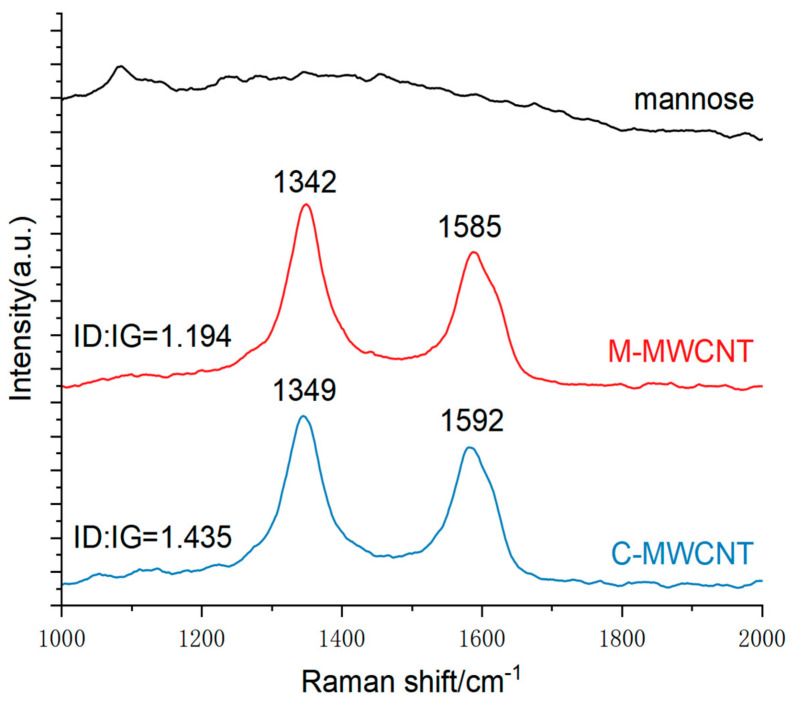
Raman spectra of the C-MWCNT, M-MWCNT, and mannose.

The Raman spectra are shown in Figure 4. The peak at 1342–1349 cm^−1^ corresponds to the D peak produced by disorder mode in C-MWCNT, i.e., the SP3 hybrid carbon atom peak at the defect position in the C-MWCNT skeleton. The G peak at 1585–1592 cm^−1^ is produced by the vibration of SP2 hybrid carbon atoms in MWCNT. This result indicates that compared to the positions of D (1342 cm^−1^) and G (1585 cm^−1^) in C-MWCNT, that of D (1349 cm^−1^) and G (1592 cm^−1^) were shifted in M-MWCNT. The ID/IG ratio of C-MWCNT and M-MWCNT was 1.194 and 1.435, respectively. Thus, modification of MWCNT with mannose increased the ID/IG ratio.

### 3.2. M-MWCNT Cytotoxicity on Macrophages

**Figure 5:**
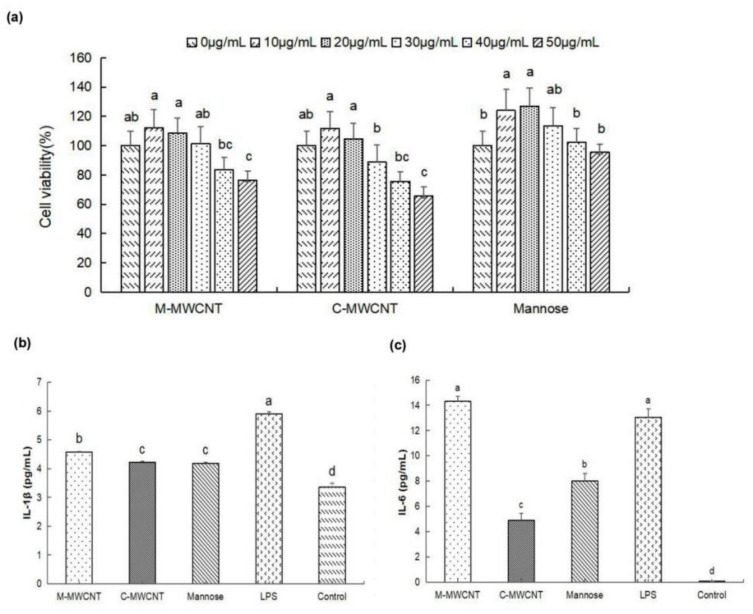
The effects of M-MWCNT on macrophage proliferation and cytokines secretion. (**a**). M-MWCNT, C-MWCNT, and mannose cytotoxicity on macrophages (**a**). IL-1β secretion levels of macrophages (**b**). IL-6 secretion levels of macrophages (**c**). Significant differences are indicated by different letters (a–f). The data are presented as the mean ± SD (*n* = 3) analyzed by one-way ANOVA and then Duncan’s multiple-range tests. Different letters (a, b, c, and d) above each group of bars indicate statistically significant differences (*p* < 0.05).

Based on the cytotoxicity analysis, we found that 30 µg/mL was the highest concentration of M-MWCNT and C-MWCNT that was not cytotoxic to macrophages. At 50 µg/mL of M-MWCNT and C-MWCNT, the survival rates of macrophages decreased significantly (*p* < 0.05). Furthermore, 30 µg/mL was the highest concentration of mannose that was not cytotoxic to macrophages (Figure 5a).

### 3.3. Effects M-MWCNT on Cytokine Secretion of Macrophages

As illustrated in Figure 5b,c, the supernatant IL-1β concentration was significantly higher in the M-MWCNT group than in the C-MWCNT, mannose, OVA, and blank groups (*p* < 0.05). Moreover, the IL-6 concentration was significantly higher in the M-MWCNT and LPS groups than in the other group (*p* < 0.05). Compared to the C-MWCNT group, the IL-6 concentration was markedly higher in the M-MWCNTs group.

### 3.4. The Ultrastructure and Confocal Morphology of Macrophages

**Figure 6:**
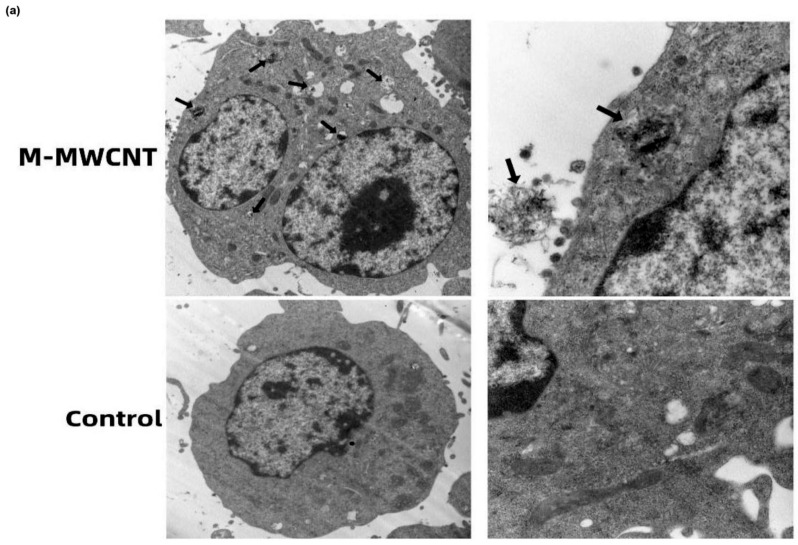

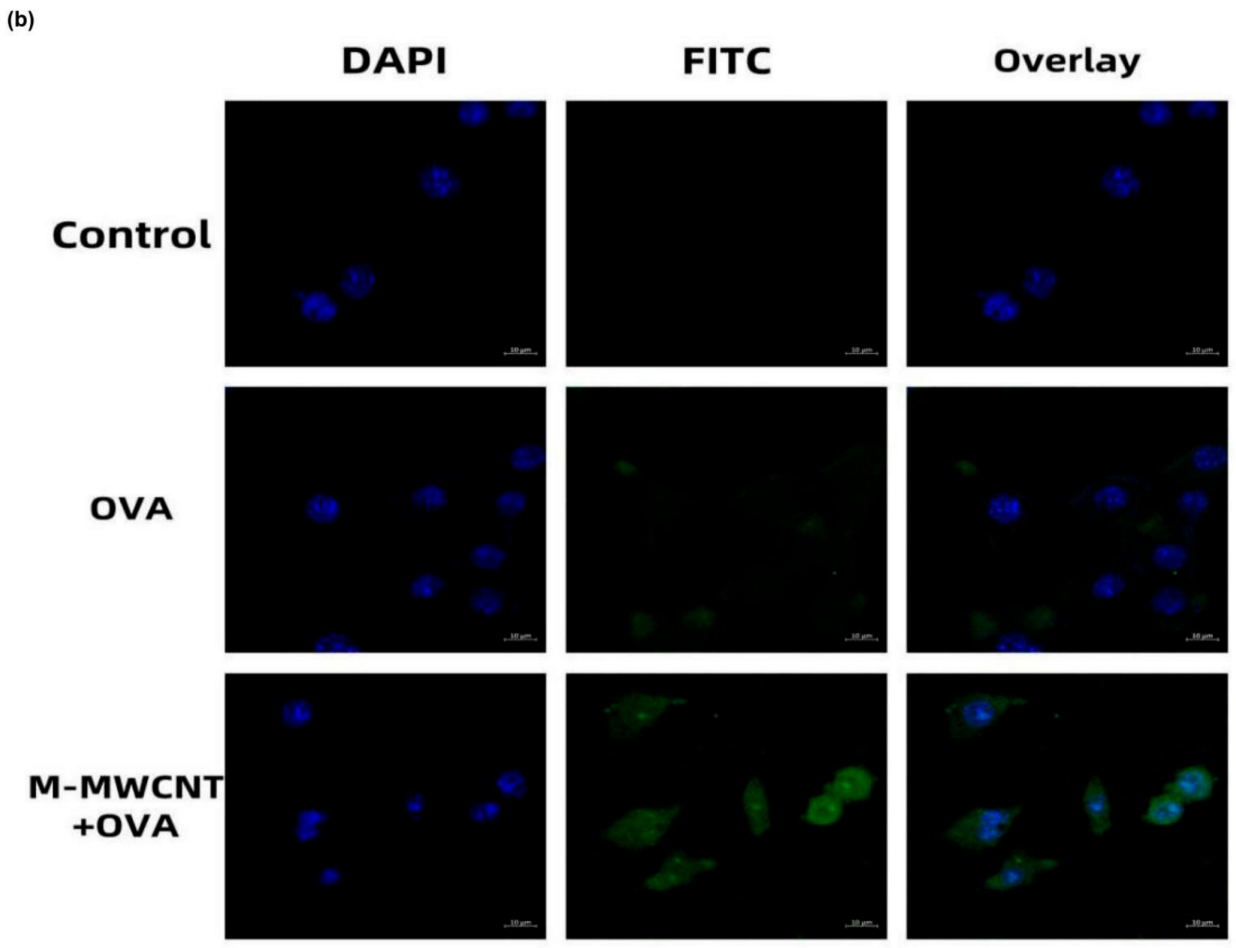
CLSM and TEM image of M-MWCNT uptake by RAW267.4. (**a**) TEM micrographs of M-MWCNT (indicated by arrow) internalized by macrophages. M-MWCNT was co-cultured with macrophages for 12 h and then the collected cells were immediately fixed with 2.5% glutaraldehyde. The fixed cells were sectioned and observed by TEM. (**b**) CLSM image of FITC-OVA uptake by RAW267.4. Macrophages were cultured with M-MWCNT+FITC-OVA or FITC-OVA for 24 h. The slides were fixed and stained with DAPI as indicated by blue fluorescence, while green fluorescence indicates FITC-OVA. The cells were observed and photographed with a laser confocal scanning microscope.

To examine the phagocytic ability of macrophages for M-MWCNT, the ultrastructural changes and CNTs distribution in macrophages were observed by TEM. We found that the control group of macrophages was nearly round, having an intact cell structure, several mitochondria, and other organelles. However, after 24 h of co-culture with M-MWCNT, the number of intracellular vesicles increased in macrophages, and a large number of CNTs were phagocytized that were transported to various cellular regions, including the nucleus (Figure 6a).

To evaluate the antigen internalization effect of M-MWCNT, the antigen distribution in macrophages was investigated by CLSM. After DAPI staining, the macrophage nucleus turned blue and appeared round under a laser confocal microscope, while FITC-labeled OVA emitted green fluorescence. These results showed that macrophages alone were capable of FITC-OVA uptake. However, the fluorescence intensity in the M-MWCNT group was stronger than in the FITC-OVA group, indicating increased OVA uptake in the former (Figure 6b).

### 3.5. Activation and Maturation of Splenic Dendritic Cells

**Figure 7:**
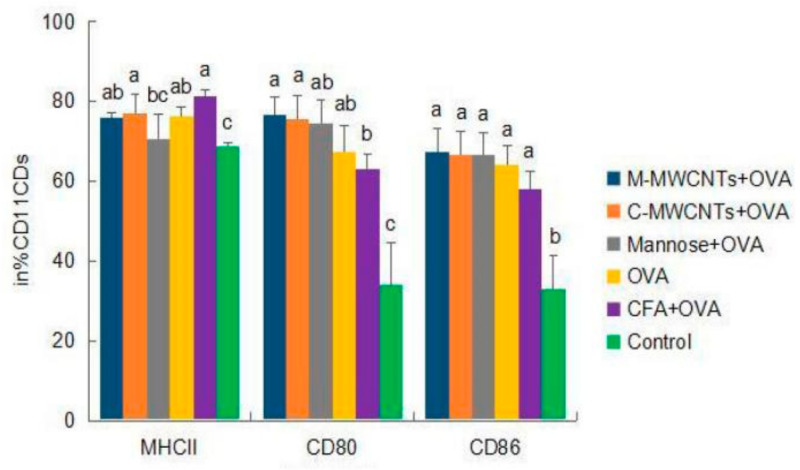
Effects of M-MWCNT on the maturation of DCs in ICR mice. The splenocytes suspension were prepared from the spleens of ICR mice 48 h after the first immunization. Point diagram analysis of dendritic cell double-stained with CD11c^+^-MHC-II^+^, CD11c^+^-CD86^+^, and CD11c^+^-CD80^+^. The percentage of CD80^+^, MHC II^+^, and CD86^+^ cells against total DCs was determined by using flow cytometry. Distribution map of CD11c^+^-MHCII^+^, CD11c^+^-CD86^+^, and CD11c^+^-CD80^+^ co-expressing cells in Supplementary Materials. The data are presented as the mean ± SD (*n* = 3) analyzed by one-way ANOVA and then Duncan’s multiple-range tests. Different letters (a, b, c, and d) above each group of bars indicate statistically significant differences (*p* < 0.05).

Forty-eight hours after primary immunization, the MHC-II^+^ frequency of dendritic cells was significantly higher in the M-MWCNT group than in the control group (*p* < 0.05). Moreover, the frequency of CD11c-CD80^+^ was dramatically higher than in Freund’s adjuvant and blank control groups (*p* < 0.05). Likewise, the expression frequency of CD11c^+^-CD86^+^ was too high in the M-MWCNT group than in the control group (*p* < 0.05, Figure 7).

### 3.6. The Effects of M-MWCNT on Splenocytes Proliferation

**Figure 8:**
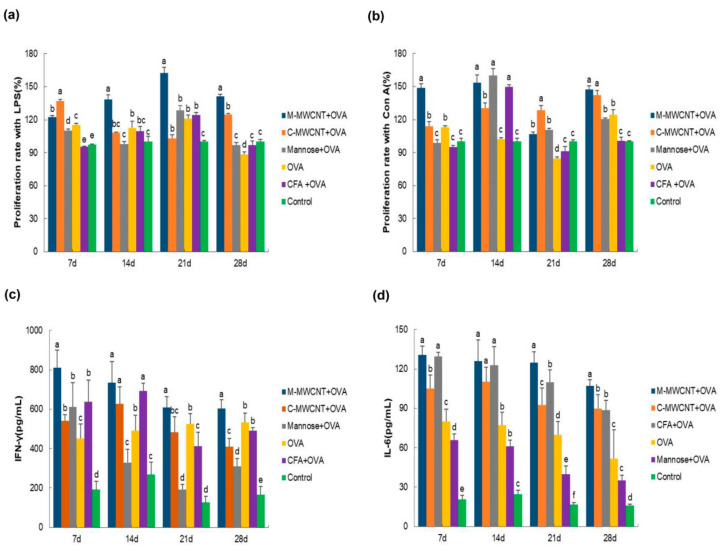
The M-MWCNT increased the proliferation of splenocytes and M-MWCNT the secretion of cytokines. The total splenocytes were isolated from the spleen of ICR mice in all groups on days 7, 14, 21, and 28 after the last immunization. The splenocyte proliferation rate (**a**,**b**) was estimated by the CCK-8 method. LPS and Con A, used as a mitogen, were co-cultured with splenocytes. The proliferation activity is represented as proliferation rate (%). The serum concentrations of IFN-γ (**c**) and IL-6 (**d**) in ICR mice were measured using ELISA. The data are presented as the mean ± SD (*n* = 3) analyzed by one-way ANOVA and then Duncan’s multiple-range tests. Different letters (a–f) above each group of bars indicate statistically significant differences (*p* < 0.05).

On days 7, 14, 21, and 28 after the primary immunization, the splenocytes were co-cultured with LPS for 48 h to investigate their proliferation rate by the CCK-8 assay. We found that on day 7, the synergistic LPS proliferation ability of lymphocytes in the M-MWCNT group was significantly higher than in the mannose, ovalbumin, and control groups (*p* < 0.05). Moreover, on days 14, 21, and 28, the proliferation rate of splenocytes was dramatically higher in the M-MWCNT group than in the other five groups (*p* < 0.05). This indicates that the proliferation of lymphocytes co-cultured with LPS was remarkably higher in the M-MWCNT group than in the C-MWCNT, OVA, mannose, or control groups (*p* < 0.05, Figure 8a).

The results of splenocytes culture with Con A for 48 h are shown in Figure 8b. On days 7, 21, and 28, the lymphocyte proliferation rate was significantly higher in the M-MWCNT group than in any other group (*p* < 0.05). On day 14, lymphocyte proliferation in the M-MWCNT group was significantly higher than in the C-MWCNT, control, and OVA groups (*p* < 0.05).

### 3.7. The Effects of M-MWCNT on the Serum Concentration of IL-6 and IFN-γ

On days 7, 21, and 28 after the final immunization, the serum concentration of IFN-γ in the M-MWCNT group was significantly higher than in any of the five groups (*p* < 0.05). On day 14, IFN-γ levels in the M-MWCNT, C-MWCNT, and Freund’s adjuvant groups were significantly elevated than in the mannose, OVA, and control (blank) groups (*p* < 0.05, Figure 8c). Moreover, the IL-6 serum concentration was remarkably higher in the M-MWCNT group than in any of the other four groups, excluding the mannose group on day 7 (*p* < 0.05). On day 14, IL-6 levels in the M-MWCNT, C-MWCNT, and mannose groups were significantly higher compared to the OVA, Freund’s adjuvant, and control groups (*p* < 0.05). Likewise, on day 21, IL-6 levels in the M-MWCNT group were dramatically higher than in any other group. On day 28, IL-6 levels in the M-MWCNT group were dramatically higher than in the other group (*p* < 0.05). There is no significant difference between the C-MWCNT group and CFA-OVA group, and there is no significant difference between the OVA group and mannose +OVA group (*p* > 0.05, Figure 8d).

### 3.8. OVA-Specific IgG1, IgG, IgG2a, and IgG2b Serum Levels in Mice

**Figure 9:**
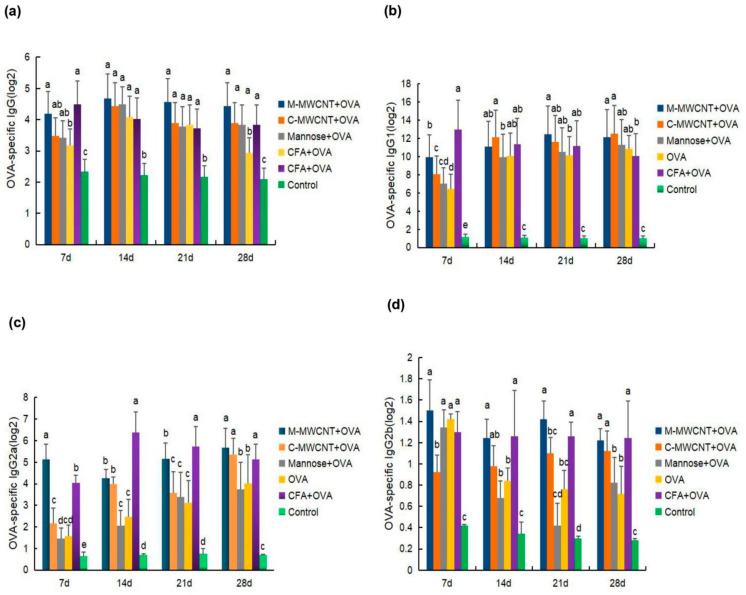
Effects of M-MWCNT on serum levels of IgG (**a**), IgG1 (**b**), IgG2a (**c**), and IgG2b (**d**) in ICR mice. Serum samples of ICR mice collected on days 7, 14, 21, 28 after the first immunization were analyzed by ELISA. The data are presented as the mean ± SD (*n* = 3) analyzed by one-way ANOVA and then Duncan’s multiple-range tests. Different letters (a, b, c, and d) above each group of bars indicate statistically significant differences (*p* < 0.05).

To evaluate the humoral immune response to the M-MWCNT antigen presentation system, the specific mice serum levels of IgG and its subclasses (IgG1, IgG2a, and IgG2b) were determined on days 7, 14, 21, and 28 after the final immunization. We found that OVA-specific IgG levels were significantly higher in the M-MWCNT group than in the control group (*p* < 0.05; Figure 9a). Serum IgG levels in the M-MWCNT group were similar to the CFA group (*p* > 0.05). This indicates that M-MWCNT induced serum IgG levels were similar to Freund’s adjuvant group.

On day14, IgG1 levels in the M-MWCNT group were significantly higher than in the mannose and control groups (*p* < 0.05), On day 21 after the final immunization, IgG1 levels in the M-MWCNT group were significantly higher than in any of the other five experimental groups. Likewise, on day 28, IgG1 levels in the mannose CNT group were remarkably higher than in the control (blank) and CFA groups (Figure 9b).

On day 7, M-MWCNT significantly increased the serum IgG2a levels in immunized mice compared to the other adjuvants or control group (*p* < 0.05). On day 21, IgG2a levels in the CNT group were significantly up-regulated than in the C-MWCNT group (*p* < 0.05). On day 28 after the final immunization, the IgG2a levels in the M-MWCNT group were markedly higher than in the Freund’s adjuvant group (Figure 9c).

As shown in Figure 9d, on day 7 after the last immunization, the IgG2b levels in the M-MWCNT group were dramatically higher than in the C-MWCNT and blank control groups (*p* < 0.05). Likewise, on day 14, the IgG2b levels in the M-MWCNT group were significantly higher than in the mannose, OVA, and control groups (*p* < 0.05). On days 21 and 28, the IgG2b levels in the M-MWCNT group were significantly elevated than in the mannose, blank, and OVA groups (*p* < 0.05).

### 3.9. In Vivo Biodistribution Analysis

**Figure 10:**
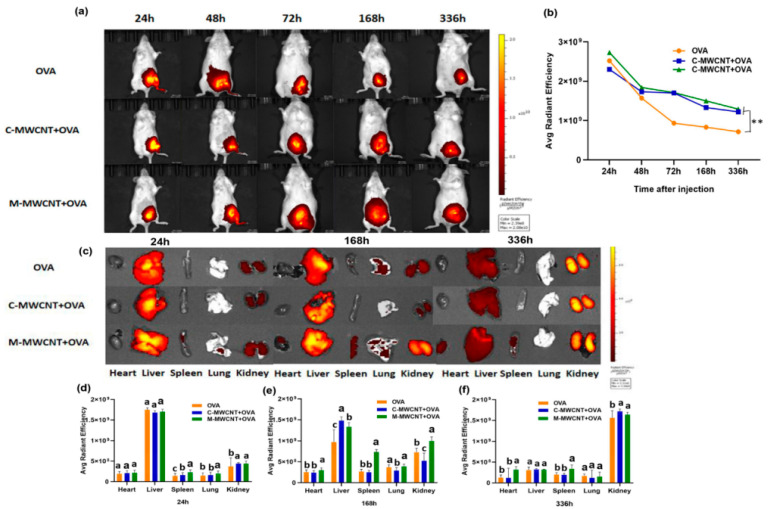
Living animal imaging on ICR mice. The OVA was labeled using a Cy5.5 fluorescent dye, and the mice were immunized with M-MWCNT+OVA, C-MWCNT+OVA, or OVA. The living animal imaging and fluorescence intensities in the mice at 24, 48, 72, 168, and 336 h after injection was detected by an in vivo optical imaging system (**a**,**b**), ** *p* < 0.01. Direct imaging and fluorescence intensities of the spleen, liver, heart, kidney, and lung of injected mice were detected on 24, 168, and 336 h after injection (**c**–**f**). Different letters (a–c) above each group of bars indicate statistically significant differences (*p* < 0.05).

The in vivo release and distribution of M-MWCNT+OVA and C-M-MWCNT+OVA in ICR mice were determined by both in vivo body imaging and direct imaging of the organs collected from ICR mice. As shown in Figure 10a,c, the fluorescence intensity of cy5.5-labeled OVA at the injection site was evident at 24 h post-injection and then gradually attenuated with time. Meanwhile, post-48 h, the fluorescence intensity of mice within the OVA and C-MWCNT+OVA groups diminished notably, while the fluorescence intensity in the M-MWCNT+OVA group decreased slowly. In the OVA group, the fluorescence intensity at the injection site was severely attenuated at 336 h post-injection, suggesting that the majority of OVA was released from the thigh at the injection site at that time. Moreover, compared to the OVA group, the fluorescence intensity at the injection site in the mice within the M-MWCNT+OVA group was remarkably higher at 336 h. The fluorescence images of the harvested tissues (spleen, liver, heart, kidney, and lung) of injected mice at different time points were determined according to the method described above. As illustrated in Figure 10b,d, the fluorescence intensity within the spleen at 24 h post-injection was observed in the M-MWCNT+OVA group and suggested that the OVA was successfully presented to the secondary lymphoid organ. In addition, the fluorescence intensity in the spleens within the M-MWCNT+OVA group was significantly higher than the spleens within the C-MWCNT+OVA and OVA groups at 24 h, 168 h, and 336 h (*p* < 0.05). In all three groups, evident fluorescence was found within the kidney at 24 h, 168 h, and 336 h post-injection, and the fluorescence intensity increased gradually with time. The fluorescence intensity within the kidneys of the M-MWCNT+OVA group was significantly higher than the intensities in the OVA and C-MWCNT+OVA groups at 24 h, 168 h, and 336 h (*p* < 0.05). This indicated that the main excretion pathway for M-MWCNT-OVA was probably through the kidney. In all three groups, the intensity of fluorescence was detected in the liver at 24 h and 168 h post-injection, which decreased at 336 h post-injection. The fluorescence intensity within the livers of the M-MWCNT+OVA group was significantly higher than the intensities for the C-MWCNT+OVA and OVA groups at 336 h (*p* < 0.05). In the M-MWCNT+OVA group, the fluorescence intensity was determined in the lung at 24 h and 168 h post-injection, which decreased at 336 h post-injection. The fluorescence intensity observed in hearts was found at 168 h and 336 h post-injection in both the M-MWCNT+OVA/C-MWCNT+OVA groups.

### 3.10. In Vivo Histological Toxicity Analysis of M-MWCNT+OVA

To examine the toxicity of M-MWCNT+OVA in vivo, tissues from major organs (heart, liver, spleen, kidney, and lung) were harvested from each group, and histological analysis was performed. The results in Supplementary Materials, hematoxylin and eosin (H&E) staining suggested that tissue morphology was comparable between mice treated with C-MWCNT+OVA, mannose+OVA, OVA, CFA+OVA, and saline. These results indicated that M-MWCNT+OVA and C-MWCNT+OVA did not cause any obvious toxicity in vivo. Thus, M-MWCNT+OVA exhibited excellent biocompatibility in vivo and showed no toxic effects when used as a drug delivery system.

## 4. Discussion

In recent years, CNTs have gained much attention in the field of applied materials science, and several studies have shown that functional modifications can greatly reduce their biotoxicity [22,31,32]. C-MWCNT can be modified with mannose by the carbodiimide catalysis method by activating the surface carboxyl group of CNT. In this reaction, EDC is added to the CNT solvent. Then, the activated surface carboxyl group of the CNT forms an EDC-CNT intermediate. Next, pyridine is added for catalytic esterification, and the EDC intermediate reacts with the -OH of mannose to yield M-MWCNT. The reaction conditions of this method are mild and controlled excluding the influence of excessive organic matter on subsequent experiments [33].

It is now well established that the hollow tubular structure, delocalized pi-electron cloud, and surface depression of CNTs significantly improve their surface protein binding, and thereby CNTs exhibit strong antigen adsorption ability [16,34]. The antigen (OVA) adsorption capacity and aqueous stability of M-MWCNT complexes were tested by the BCA assay kit and zeta potential value. We found that M-MWCNT exhibited strong antigen adsorption capacity. Moreover, after antigen adsorption, the absolute value of the zeta point significantly increased to 29.1 (mV). In general, particles with a surface potential of >15 (mV) are fairly stable in the solvent [20,35]. Therefore, M-MWCNT seems to have good stability in aqueous solution.

Macrophages, a kind of widely distributed APC cell, play a crucial function during the immune response and antigen presentation. These create an important bridge between innate and adaptive immunity. The physiological function of macrophages is to clear invading pathogens by phagocytosis [36]. Cytokines secretion is another important manifestation of cells participating in immune responses. The secretion of IL-1β and IL-6 has been closely associated with macrophages. IL-1β, originally identified as the major febrile endogenous pyrogen, has been shown to impart various responses in infection, injury, and immune challenges [37,38]. Likewise, IL-6, mainly produced by Th2 cells, mononuclear macrophages, fibroblasts, and vascular endothelial cells, is involved in specific cellular and humoral immune response by participating in B cell proliferation and differentiation, T cell activation, and immunoglobulin secretion [39,40]. In our study, we found that M-MWCNT can simultaneously promote IL-1β and IL-6 secretion from macrophages in vitro, indicating its efficiency as an antigen delivery system.

Th1/Th2 cell dichotomy is the hallmark of the cellular immune response which can be estimated from their in vivo levels [41,42]. Qu et al. (2012) demonstrated that M-MWCNT promoted serum cytokines (IL-1 β and IL-6) levels in mice [43]. Ma et al. investigated the effects of 21 different kinds of CNTs on the immune system that were modified for length, diameter, and functional groups. They found that almost all CNTs triggered significant immune responses, and the original CNT and C-MWCNT could also promote the levels of IL-2 [44]. Notably, in this study, we obtained similar results. M-MWCNT promoted the release of IFN-γ (Th1) and IL-6 (Th2) to balance the Th1/Th2 immune response. Moreover, with an increase in days after the last immunization, the cytokines levels in the M-MWCNT group decreased more slowly than in any other experimental group, showing a long-term immune response from M-MWCNT.

Many studies have found that CNTs can be taken up by mammalian cells through active and passive transport leading to distribution in the cytoplasm, endosomes, and lysosomes [45,46]. In our study too, the cellular distribution of M-MWCNT was broadly similar to previous studies. Wenjun Yao et al. demonstrated that mannose-modified chitosan nanoparticles increased the phagocytosis of FITC-pGRP by macrophages [47]. Qingming Xia conducted an in vitro cytological evaluation of MHR/CpG nanoimmune preparations and found that RAW264.7 cells exhibited significantly higher uptake for the loaded CpG nanoimmunization system [48]. Here, we found that after phagocytosis of M-MWCNT-carrying FITC-OVA, macrophages showed stronger fluorescence than the control FITC-OVA group, suggesting improved antigen delivery efficiency of M-MWCNT via phagocytosis.

DCs are highly specialized antigen-presenting cells (APCs) that are involved in adaptive and innate immune responses [49]. Notably, mannose receptors are highly expressed on dendritic cells and macrophages [22,50]. In our study, we found that mannose-modified CNTs significantly promoted the expression of MHC-II antigen-presenting molecules, CD80, and CD86 co-stimulatory factors. Since the presentation of peptide antigens by MHC molecules of dendritic cells (APC) is essential for enhancing the immune response, it is generally considered that an efficient antigen delivery vaccine vector would be most useful [51]. Our results suggest that M-MWCNT effectively stimulated dendritic cells’ maturation and differentiation and improved their ability of phagocytosis, processing, and antigen presentation.

The activation, proliferation, and clonal expansion of antigen-specific lymphocytes are important signs of adaptive immune response to pathogens [52]. The lymphocyte proliferative response varies with the type of mitogen. To investigate the immune response of M-MWCNT, we stimulated splenocytes using LPS and Con A. We found that M-MWCNT promoted the proliferation of T and B lymphocytes within 28 days after the final immunization.

B cells-produced IgG is the main immune molecule of the humoral immune response [53,54]. The serum levels of OVA-specific IgG and IgG subclasses (IgG1, IgG2a, and IgG2b) were used to evaluate the humoral immune response of the M-MWCNT antigen delivery system. We found that M-MWCNT significantly promoted the humoral immune response by aggravating the production of OVA-specific IgG and IgG subclasses.

The sustained release of antigen is a crucial characteristic in the evaluation of novel antigen delivery systems since the sustained and slow release of antigen is closer to the process of natural acute infection by pathogens. Live-animal imaging can reflect the characteristics of antigen released from the injection site at different times, and direct imaging of the tissues and organs can certainly demonstrate the distribution of fluorescently labeled antigen [55]. In the present study, the intensity of fluorescence OVA at the thigh injection site was obvious at 24 h post-injection and then gradually diminished with time, with such fluorescence intensity at the injection site for mice in the OVA group decreasing significantly post-72 h, while fluorescence intensity for mice in the M-MWCNT+OVA group was notably higher than for mice in C-MWCNT+OVA and OVA groups at 336 h, suggesting that the M-MWCNT+OVA—as a delivery system—can effectively control the sustained release of OVA in thigh injection sites. Lymph organs play a crucial role in initiating adaptive immunity. Antigens are processed and presented by APCs, which can migrate to secondary lymph organs where they present antigens to T or B cells and activate them, further improving immune responses. In the current study, the fluorescence intensity of OVA for the spleen in the M-MWCNT+OVA group was stronger than in the spleen within C-MWCNT+OVA and OVA groups at 24 h, 168 h, and 336 h. This suggested that the antigens were presented to the secondary lymphoid organ successfully, and M-MWCNT can be targeted to lymph organs and prolong the retention time of OVA at the injection site and lymph organs, contributing to inducing an effective and sustained immune response. The lung distribution data demonstrated that M-MWCNT+OVA was observed in the lung within 24 and 168 h and diminished at 336 h, indicating that a small amount of M-MWCNT+OVA was distributed within the lung and was rapidly excreted. The heart distribution results showed that the fluorescence intensity in the heart increased gradually with time, which could be due to the slow-release effect of M-MWCNT. In addition, compared with the C-MWCNT+OVA and OVA groups, a higher distribution was observed within the liver and kidney for the M-MWCNT+OVA group, revealing that the main excretion pathway of M-MWCNT and C-MWCNT was probably the kidney/liver pathway. Many other studies have demonstrated that MWCNT—as a drug delivery system—was mainly distributed in the liver, lung, and spleen and excreted from the liver and kidney, which was consistent with our experimental results [55,56].

## 5. Conclusions

In summary, we successfully synthesized stable mannose–CNT complexes catalyzed by EDC/DMAP. M-MWCNT promoted IL-1β and IL-6 secretion from macrophages and efficiently improved phagocytosis leading to distribution in the cytoplasm, phagosomes, and lysosomes. This significantly promoted antigen internalization and up-regulated the cellular immune response in vitro. In vivo, M-MWCNT induced the maturation of dendritic cells, proliferation of T and B lymphocytes, and M-MWCNT stimulated Th1/Th2 cytokines. Besides, M-MWCNT+OVA induced a long-term antigen-specific IgG and IgG subclass antibody production due to the release of OVA which was prolonged as a result of MWCNT mannose modification. Taken together, M-MWCNT can be an efficient targeted antigen delivery system with low toxicity.

## Data Availability

Not applicable.

## Supplementary Materials

The following supporting information can be downloaded at: https://www.mdpi.com/article/10.3390/ijms23084239/s1.
