# Correction: Feng et al. Mannose Receptor-Mediated Carbon Nanotubes as an Antigen Delivery System to Enhance Immune Response Both In Vitro and In Vivo. *Int. J. Mol. Sci.* 2022, *23*, 4239

**DOI:** 10.3390/ijms26093975

**Published:** 2025-04-23

**Authors:** Haibo Feng, Yangyang Feng, Lang Lin, Daiyan Wu, Qianqian Liu, Hangyu Li, Xinnan Zhang, Sheng Li, Feng Tang, Ziwei Liu, Linzi Zhang

**Affiliations:** 1 College of Animal Husbandry and Veterinary Medicine, Southwest Minzu University, Chengdu 610041, China; f1733678933@126.com (Y.F.); wdx1063196822@126.com (D.W.); 15892603728@163.com (Q.L.); li1998hangyu@163.com (H.L.); n1547187363@163.com (X.Z.); listen699@163.com (S.L.); tangfeng1719556170@163.com (F.T.); z17393117167@163.com (Z.L.); zlz754130837@163.com (L.Z.); 2 Key Laboratory of Ministry of Education and Sichuan Province for Qinghai-Tibetan Plateau Animal Genetic Resource Reservation and Utilization, Chengdu 610041, China; 3 College of Veterinary Medicine, Southwest University, Rongchang, Chongqing 402460, China; umbrella_fj@163.com

In the original publication [1], there was a mistake in the Figure 10. Living animal imaging on ICR mice. Due to confusion regarding the experimental result number, the live-animal imaging pictures of group C-MWCNT+OVA at 168 h in Figure 10c were incorrectly uploaded. We replaced and corrected the affected pictures. The correct figure appears below. The authors state that the scientific conclusions are unaffected. This correction was approved by the Academic Editor. The original publication has also been updated.

**Figure 10:**
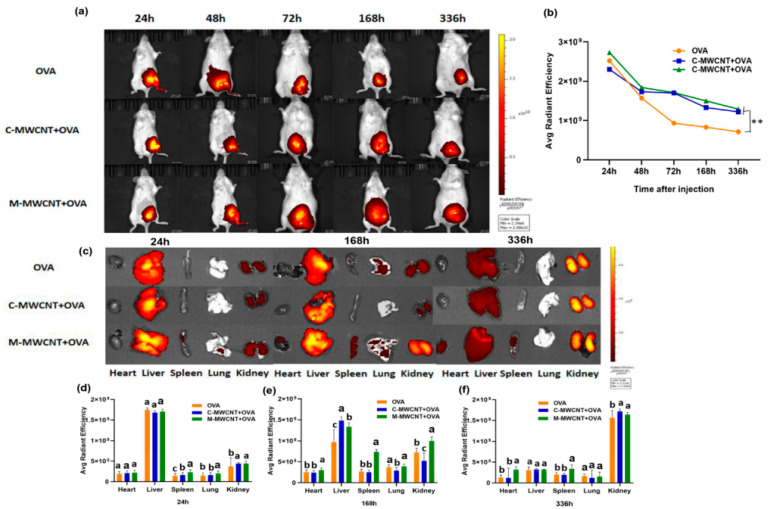
Living animal imaging on ICR mice. The OVA was labeled using a Cy5.5 fluorescent dye, and the mice were immunized with M-MWCNT+OVA, C-MWCNT+OVA, or OVA. The living animal imaging and fluorescence intensities in the mice at 24, 48, 72, 168, and 336 h after injection was detected by an in vivo optical imaging system (**a**,**b**), ** *p* < 0.01. Direct imaging and fluorescence intensities of the spleen, liver, heart, kidney, and lung of injected mice were detected on 24, 168, and 336 h after injection (**c**–**f**). Different letters (a–c) above each group of bars indicate statistically significant differences (*p* < 0.05).
