# Supplementary figures and images for: Mannose Receptor-Mediated Carbon Nanotubes as an Antigen Delivery System to Enhance Immune Response Both In Vitro and In Vivo
Source: Int J Mol Sci. 2022 Apr 11;23(8):4239. doi: 10.3390/ijms23084239 (PMC9030879; doi:10.3390/ijms23084239)

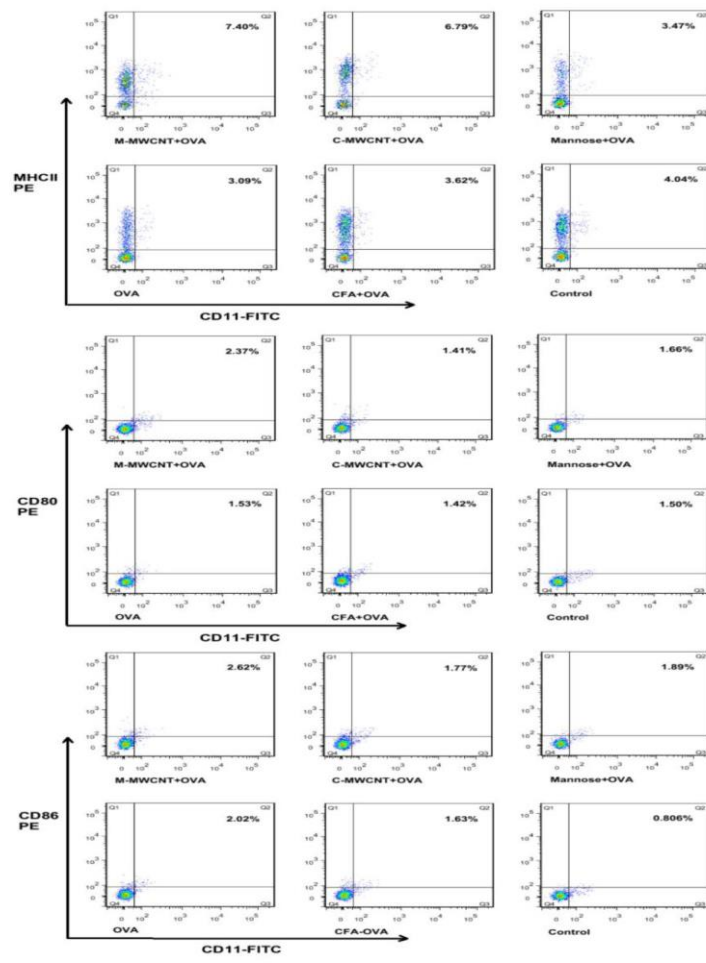

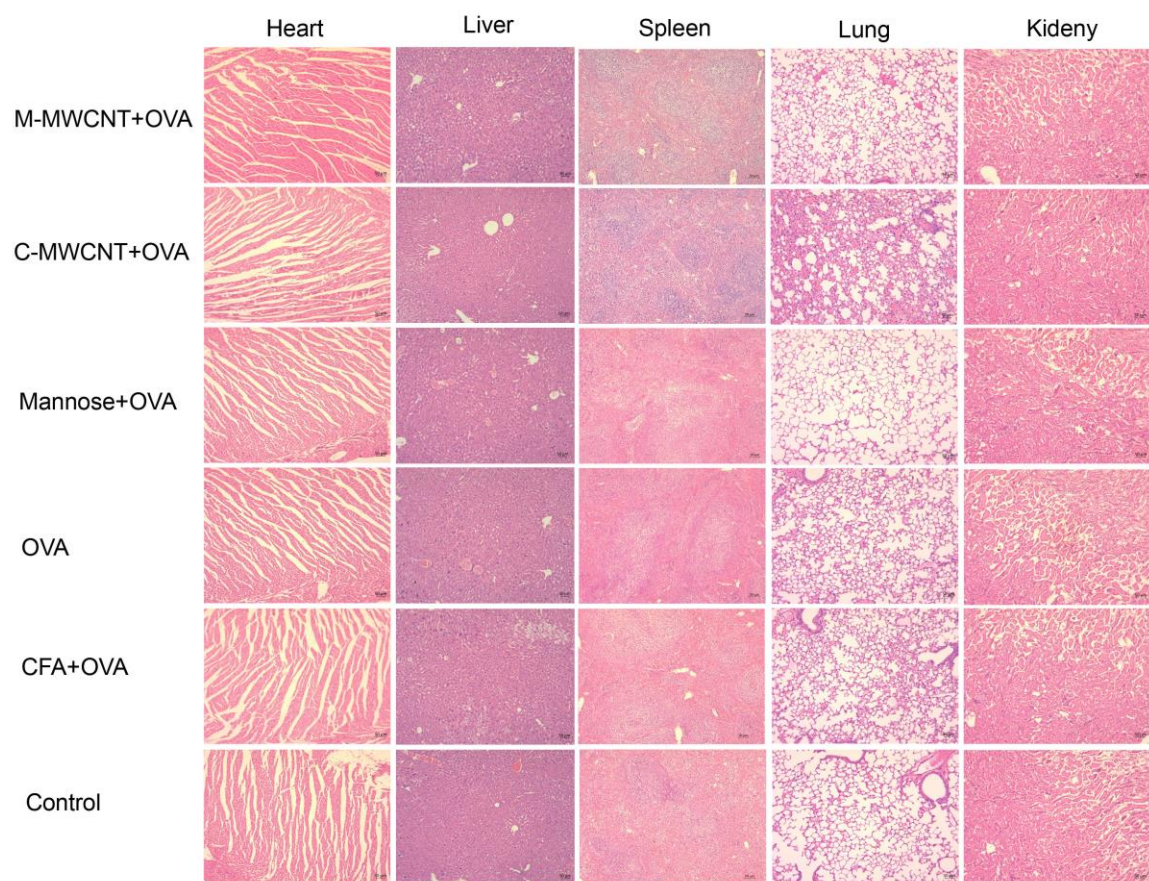

Supplement: Supplementary file 1 [file ijms-23-04239-s001.zip › ijms-1610317-supplementary.pdf]
